# Novel Components of the Stress Assembly Sec Body Identified by Proximity Labeling

**DOI:** 10.3390/cells12071055

**Published:** 2023-03-30

**Authors:** Chujun Zhang, Elisavet Kalaitsidou, J. Mirjam A. Damen, Rianne Grond, Catherine Rabouille, Wei Wu

**Affiliations:** 1 Hubrecht Institute of the KNAW and UMC Utrecht, 3584 CT Utrecht, The Netherlands; 2 Singapore Immunology Network (SIgN), Agency for Science, Technology and Research (A*STAR), 8A Biomedical Grove, Immunos, Singapore 138648, Singapore; 3 Department of Pharmacy, National University of Singapore, Singapore 117543, Singapore; 4 Biomolecular Mass Spectrometry and Proteomics, Bijvoet Center for Biomolecular Research and Utrecht Institute of Pharmaceutical Sciences, Utrecht University, Padualaan 8, 3584 CH Utrecht, The Netherlands; 5 Department of Biological Science of Cells and Systems, UMC Groningen, 9713 AV Groningen, The Netherlands

**Keywords:** Sec body, APEX2, mass spectrometry, early secretory pathway, stress granules

## Abstract

Sec bodies are membraneless stress-induced assemblies that form by the coalescence of endoplasmic reticulum exit sites (ERES). Through APEX2 tagging of Sec24AB, we biotinylated and identified the full complement of Sec body proteins. In the presence of biotin-phenol and H_2_O_2_ (APEX on), APEX2 facilitates the transfer of a biotin moiety to nearby interactors of chimeric Sec24AB. Using this unbiased approach comparing APEX on and off (−H_2_O_2_) conditions, we identified 52 proteins specifically enriched in Sec bodies. These include a large proportion of ER and Golgi proteins, packaged without defined stoichiometry, which we could selectively verify by imaging. Interestingly, Sec body components are neither transcriptionally nor translationally regulated under the conditions that induce Sec body formation, suggesting that incorporation of these proteins into granules may be driven instead by the aggregation of nucleating proteins with a high content of intrinsically disordered regions. This reinforces the notion that Sec bodies may act as storage for ERES, ER and Golgi components during stress.

## 1. Introduction

The intracellular content is compartmentalized through the formation of either membrane-bound compartments sealed by a lipid bilayer [1], or membraneless organelles [2]. The latter are formed by phase separation, a process by which diffuse macromolecules coalesce into a mesoscale structure [3,4]. Although a number of membraneless organelles are stable in growing cells and are critical to their physiology (such as the nucleolus) [5], others appear to form in response to cellular stress. For instance, ER and oxidative stress can lead to the formation of reversible stress granules [3,6,7,8] that contain specific RNA binding proteins [9] and a large collection of RNAs [10] that appear to be stored in these structures during the period of stress.

Interestingly, stress granules can also form in stressed Drosophila S2 cells [11,12], along with other stress assemblies that are not RNA-based. In this regard, we have shown that amino-acid starvation combined with salt stress (in the Krebs Ringer Bicarbonate buffer, KRB) can trigger Sec body formation [12,13].

Sec bodies form around components functioning at ER exit sites (ERES), where proteins are destined for the plasma membrane, i.e., the extracellular medium, and nearly all membrane compartments of the cell exit the ER and transit to the Golgi apparatus and correct downstream compartments. ERES are characterized by COPII-coated vesicles of 70 nm in diameter [14,15] that are concentrated near an ER cup-shaped membrane [16,17]. COPII coat formation is regulated and requires a defined set of proteins. Sec12 is a key transmembrane protein in this process and acts as a GTP-exchange factor for the small GTPase Sar1. Once in its GTP-bound form, Sar1 is inserted in the ER membrane and recruits the two COPII subunits Sec23/Sec24 that together form the inner COPII coat [18]. The outercoat Sec13/31 is then recruited. This coat assembly is concomitant with the budding of vesicles at the ER cup-shaped membrane, leading to the formation of COPII-coated vesicles [19]. Importantly, optimal COPII coat assembly also requires a large protein Sec16 that has been shown to interact with all COPII subunits [20]. This is exemplified by the effect of Sec16 mutation or loss of function in many species that leads to a severe impairment in trafficking through the secretory pathway [16,21,22,23,24,25,26,27]. Interestingly, it has been reported that COPII also has a role at ER exit sites to regulate the entry of cargo into uncoated structures [28,29].

Sec bodies are non-membrane bound compartments that are quickly reversible upon stress relief and display features of liquid-like droplets [12], consistent with their formation by phase separation. This process has been shown to be driven by specific proteins that when depleted prevent condensation of a given structure. These drivers have been shown to contain a large number of low-complexity sequences that are often intrinsically disordered (Banani et al., 2016). In this regard, depletion of the Sec24AB isoform [12] and Sec16 [30] from Drosophila S2 cells prevents Sec body formation. Interestingly, these two proteins contain a large proportion of disordered regions that are concentrated in the N-terminal sequence in Sec24AB and throughout the protein in Sec16 [12]. Importantly, membraneless organelles also contain many client proteins that passively coalesce. Interestingly, these clients also often contain disordered regions that are thought to help their recruitment [31].

To date, immunofluorescence of endogenous and tagged proteins has revealed that Sec bodies contain Sec16 as a key marker, and most of the COPII subunits Sec24AB, Sec24CD, Sec23 and Sec31 but not Sar1 [12]. In addition, few Golgi proteins appear to overlap with these structures. However, the rest of the Sec body composition is still largely unclear. Here, we aim to identify additional Sec body components using APEX2 proximity labeling [32]. APEX is a proximity ligation and affinity MS method that depends on a biotin ligase linked to a bait protein, to biotinylate surrounding protein interactors. In the presence of biotin-phenol and H_2_O_2_, APEX facilitates the transfer of a biotin moiety to nearby proteins. When the APEX is fused to a protein of a given compartment, it allows the quantitative identification of other immediate proteins in the same compartment by mass spectrometry. Interestingly, the APEX2 strategy was successfully applied to map the components of stress granules in mammalian cells [33,34].

To identify Sec body components, we tagged the COPII subunit Sec24AB with APEX2 and GFP. This chimera was successfully incorporated into Sec bodies in KRB stressed S2 cells. This allowed the identification of 52 proteins present in Sec bodies, including a large pool of ERES proteins like Sec16 and other COPII subunits, as well as components functioning at the ER and the Golgi apparatus. In line with phase separation, most of the 52 identified components contain intrinsically disordered regions that are proposed to contribute to the coalescence [35,36]. Importantly, these components are neither regulated transcriptionally nor translationally, suggesting a re-location to Sec bodies that likely could act as storage during the period of stress.

## 2. Materials and Methods

### 2.1. Cell Culture and Amino Acid Starvation

Drosophila S2 cells (R69007, Thermo Fisher Scientific, Waltham, MA, USA) were cultured in Schneider’s medium (SCH, S0146; Sigma, St. Louis, MO, USA) supplemented with 10% insect-tested fetal bovine serum (F4135; Sigma, St. Louis, MO, USA) at 26 °C. S2 cells (between passages 5 and 18) were pelleted at 200 g in a microfuge for 3 min, washed once in fresh Schneider’s medium, and diluted to 106 mL. 1 mL of cell suspension was plated per well in a 12-well plate containing coverslips. Cells were allowed to attach for 1.5 h before starting the treatment. Amino acid starvation was performed in Krebs Ringers bicarbonate buffer (KRB) comprising 0.7 mM NaH_2_PO_4_, 15 mM NaHCO_3_ (sodium bicarbonate, BIC), 120.7 mM NaCl, 4.53 mM KCl, 0.5 mM MgCl and 10 mM glucose at pH 7.4 as described previously [12,13].

### 2.2. Molecular Cloning and Transfection

To generate pMT-Sec24AB-GFP-APEX2, GFP-APEX2 was amplified from the plasmid encoding Connexin43-GFP-APEX2 (44440, addgene) and cloned into pMT using the restriction enzymes BstBI and Pmel. Sec24AB was amplified from a cDNA library made from S2 cells and cloned into pMT-GFP-APEX2 using the restriction enzymes EcoRI and Apal. To generate pMT-Rox8-V5, Rox8 was amplified from a cDNA library made from S2 cells and cloned into pMT-V5 using the restriction enzymes KpnI and EcoRv. Transfection of pMT constructs were performed using Effectene transfection reagent (301425, Qiagen, Hilden, Germany) [37] for 48 h. Chimeric protein expression was induced with 1 mM CuSO_4_ for 1.5 h before incubation in KRB.

Primers used were:

GFP-APEX2 forward: 5′-CATGTTCGAACTATGGTGAGCAAGGGCG-3′,
GFP-APEX2 reverse: 5′-CATGGTTTAAACTTAGGCATCAGCAAACCC-3′
Sec24AB forward: 5′-CATGGAATTCCACCATGTCGACTTACAATCCGAACTTC-3′
Sec24AB reverse: 5′-CATGGGGCCCTTTAACCTGAGCCCGAATGT-3′
Rox8 forward: 5′-GGGATCTAGATCGGGGTACCATGGACGAGTCGCAACCG-3**’**,
Rox8 reverse: 5′-GCCACTGTGCTGGATATCTTGGGTCTGGTATTGTGGCATCG-3**’**.

### 2.3. Stable Cell Line

To generate pMT-Sec24AB-GFP-APEX2 stable cell line, pMT-Sec24AB-GFP-APEX2 was co-transfected with a plasmid encoding for pCoHygro in S2 cells using Effectene transfection reagent (Qiagen, Venlo, The Netherlands) with a 1:10 ratio of DNA to Effectene Reagent. After culturing the transfected cells in antibiotic-free medium for 2 days, the selection of the transfected cells was performed with 150 μg/mL Hygromycin B (ThermoFisher, Waltham, MA, USA). Stable S2 cells were cultured in Schneider’s medium with additional 300 μg/mL Hygromycin B.

### 2.4. Antibodies

Immunofluorescence was performed with rabbit polyclonal anti-Sec16 (1:800) [16]; mouse monoclonal anti-GM130 (4A3 at 1:500; gift from Martin Lowe) [38]; mouse monoclonal anti-Hrs (1:10, deposited by Munro, S. DSHB) [39]; guinea pig antibody dHNG2 against Homer (1:100; gift from UIrich Thomas) [40]; and rabbit polyclonal antibody against N-terminal Lasp (1:200, gift from Anne Ephrussi) [41]. For Western blotting, biotinylated proteins were detected with streptavidin-HRP conjugates (1:1000, SA10001, life technologies). As loading control, mouse monoclonal anti-α-tubulin (1:2500, T5168, Sigma-Aldrich, St. Louis, MO, USA) was used. As secondary antibodies, we used donkey anti-rabbit-IgG conjugated to Alexa Fluor 568 (1:200, A10042, Invitrogen, Waltham, MA, USA), goat anti-guinea pig-IgG conjugated to Alexa Fluor 488 (1:200, A-11073, Invitrogen, Waltham, MA, USA), donkey anti-rabbbit-IgG conjugated to Alexa Fluor 647 (1:200, A31573, Life Technologies, Waltham, MA, USA) and streptavidin conjugated to Alexa Fluor 568 (1:200, S11226, Invitrogen, Waltham, MA, USA), as well as mouse-IgG antibodies coupled to HRP (1:2000, NA934, NA931, GE Healthcare, Chicago, IL, USA).

### 2.5. Immunofluorescence

Cells were fixed with 4% paraformaldehyde in PBS (pH 7.4) for 20 min. Cells were then washed three times with PBS and subsequently quenched by incubation in 50 mM NH_4_Cl in PBS for 5 min. Followed by permeabilization with 0.11% Triton X-100 for 5 min. Thereafter, cells were washed three times in PBS and blocked in PBS supplemented with 0.5% fish skin gelatin (G7765, Sigma-Aldrich, St. Louis, MO, USA) for 20 min. Cells were then incubated with the primary antibody (in blocking buffer) for 25 min, washed three times with blocking buffer and incubated with the secondary antibody (in blocking buffer) coupled to a fluorescent dye for 20 min. Cells on the coverslip were washed twice in milliQ water and dried for 3 min on a tissue with cells facing up. Finally, each coverslip with cells was mounted with Prolong antifade medium (+DAPI, P36935, Invitrogen, Waltham, MA, USA) on a microscope slide. Samples were viewed with a Leica SPE confocal microscope using a 63× oil lens and 2× zoom. Quantification of signal overlap with Sec bodies was performed by manual counting of all 50 fields imaged. No images were excluded. 

### 2.6. Western Blotting

A total of 4 × 10^6^ cells per condition were harvested on ice and lysed in 50 mM Tris-HCl pH 7.5, 150 mM NaCl, 1% Triton X- 100, 50 mM NaF, 1 mM Na3VO4, 25 mM Na2-β-glycerophosphate, supplemented with a protease inhibitor tablet (Roche, New York, NY, USA). The lysates were cleared by centrifugation at 20,000× *g* for 20 min at 4 °C. Supernatants were collected and the protein concentration was determined with a BCA protein assay kit (ThermoFisher, Waltham, MA, USA). 50 μg of protein was mixed with 5× SDS loading dye, boiled for 5 min and separated on an 8% SDS-PAGE gel. Then, separated proteins were transferred to a polyvinylidene difluoride (PVDF) membrane. Hereafter, the PVDF membrane was blocked in TBS + 0.05% Tween-20 and 5% BSA (Sigma-Aldrich, St. Louis, MO, USA) (Blocking buffer). Primary antibodies were added to blocking buffer. After an overnight incubation at 4 °C, the membrane was washed 3 times in TBS + 0.05% Tween-20 over 45 min and incubated with secondary antibodies for 1 h at room temperature. The membrane was washed 3 times washing in TBS + 0.05%Tween-20 and developed by enhanced chemiluminescence (Bio-Rad, Hercules, CA, USA) with Image QuantTM LAS 4000.

### 2.7. APEX Proximity Labeling and Analysis of Biotinylated Proteins

After induction cells were incubated in Schneider’s or KRB for 4 h. To induce APEX activity, 500 uM biotin-phenol was added to the medium in the last 0.5 h, and 1 mM H_2_O_2_ was added for 1 min just before harvesting. Biotinylation reaction was quenched with 5 mM Trolox and 10 mM solidum L-ascorbate in PBS. After cold-PBS wash, cells were harvested by scrapping and lysed in ice-cold lysis buffer (8 M urea, 150 mM NaCl, 20 mM Tris-HCl pH 8.0, Protease Inhibitor Cocktail, 5 mM Trolox and 10 mM sodium L-ascorbate) for 30 min on ice. Lysates were cleared at 18,000× *g* for 30 min at 4 °C and the protein concentration was determined with a BCA protein assay kit (Thermo Fisher Scientific, Waltham, MA, USA). 50 uL streptavidin-coated magnetic beads (Thermo Fisher Scientific, Waltham, MA, USA) were incubated for pulldown experiments with 380 ug protein for each condition with end-to-end rotation for 2 h at room temperature.

Biotinylated proteins captured by Steptavidin-coated magnetic beads were washed 5 times with PBS, and 3 times with 50 mM ammonium bicarbonate. Reduction was performed at 20 °C for 1 h with 10 mM dithiothreitol (DTT). Alkylation was then performed at 20 °C for 0.5 h with 20 mM iodoacetamide (IAA) in the dark. Digestion was performed at 37 °C, first with 1 μg of Lys C for 4 h, then with 1 μg of Trypsin overnight. Digested peptides were cleaned up with home-made c18 STAGE tips. Peptides were analysed by LC-MS/MS on an UHPLC 1290 system (Agilent, Santa Clara, CA, USA) coupled to an Orbitrap HF-X mass spectrometer (Thermo Scientific, USA). Peptides were trapped (Dr. Maisch Reprosil-Pur C18-AQ, 3 μm, 2.5 cm × 100 μm) for 5 min in solvent A (0.1% Formic acid in water) and then separated on an analytical column (Agilent Poroshell, 120 EC-C18, 2.7 μm, 50 cm × 75 μm) using a linear gradient of solvent B (0.1% Formic acid in 80% acetonitrile). An LC gradient of 10–40% B in 115 min was used.

The mass spectrometer was operated in data-dependent mode, at a resolution of 60,000 for MS1 and 30,000 for MS2. Peptide data were acquired at 375–1600 *m*/*z* and precursor ions were accumulated for 20 ms or until a AGC target value of 3 × 10^6^ was reached. The 15 most abundant doubly and triply charged precursors were selected for fragmentation, after accumulation to the AGC target value of 1 × 10^5^ within 50 ms. HCD fragmentation was performed at 27% NCE. Dynamic exclusion time was set to 16 s.

### 2.8. Mass Spectrometry Proteome Analysis

8 million cells per condition were grown in Schneider’s and starved in KRB for 2 h and 4 h in 6 cm dishes at 26 °C. After incubation, cells were harvested cells on ice, and cell pellets were and washed twice with ice-cold PBS. Cell material was lysed by gentle vortexing in 8 M Urea in 50 mM ammonium bicarbonate supplemented with 50 μg/mL DNAse I (Sigma-Aldrich, St. Louis, MO, USA), 50 μg/mL RNAse A (Sigma-Aldrich, St. Louis, MO, USA) and 1× cOmplete EDTA-free protease inhibitor cocktail (Roche Diagnostics, New York, NY, USA). Subsequently, the lysate was cleared by centrifugation for 1 h at 18,000× *g* at 15 °C. Protein concentration was determined with the Bradford assay (Bio-Rad, Hercules, CA, USA). For each sample, 20 μg of total protein was reduced, alkylated and digested sequentially with Lys-C (1:100) and trypsin (1:75), and perfectionated pre-fractionated offline on C18 STAGE-tips. Peptides were eluted in 5 high-pH reversed phase fractions, with 11–80% acetonitrile. All samples were dried by vacuum centrifugation and reconstituted in 102% formic acid prior to LC-MS/MS analyses.

MS data was acquired with an UHPLC 1290 system (Agilent, Santa Clara, CA, USA) coupled to a Q-Exactive HF mass spectrometer (Thermo Fischer Scientific, Waltham, MA, USA). Peptides were trapped (Dr Maisch Reprosil C18, 3 μM, 2 cm × 100 μM) for 5 min in solvent A (0.1% formic acid in water) before being separated on an analytical column (Agilent Poroshell, EC-C18, 2.7 μM, 50 cm × 75 μM). Solvent B consisted of 0.1% formic acid in 80% acetonitrile. The mass spectrometer operated in data-dependent mode. Full scan MS spectra from *m*/*z* 375–1600 were acquired at a resolution of 60,000 to a target value of 3 × 10^6^ or a maximum injection time of 20 ms. The top 15 most intense precursors with a charge state of 2+ to 5+ were chosen for fragmentation. HCD fragmentation was performed at 27% normalized collision energy on selected precursors with 16 s dynamic exclusion at a 1.4 *m*/*z* isolation window after accumulation to 1 × 10^5^ ions or a maximum injection time of 50 ms. Tandem mass spectrometry (MS/MS) spectra were acquired at a resolution of 15,000. The APEX proteomics dataset has been deposited to ProteomeXchange Consortium via the PRIDE repository and can be accessed through the identifier PXD031601.

### 2.9. Mass Spectrometry Database Search

Raw files were searched using MaxQuant version 1.5.3.30 and the Andromeda search engine against the drosophila uniprot database (42,477 entries, downloaded in September 2018). Enzyme specificity was set to trypsin and up to 2 missed cleavages were allowed. Cysteine carbamidomethylation was set as fixed modification. Methionine oxidation and N-terminal acetylation were set as variable modifications. The false discovery rate (FDR) was restricted to 1% in both protein and peptide identification. For quantitative comparisons, label-free quantification (LFQ) was performed with “match between runs” enabled. Data normalization, imputation and statistics were performed with Perseus version 1.6.2.2. The data was visualized with Graphpad PRISM 8.

## 3. Results and Discussion:

### 3.1. Sec24-APEX2 Mediates Biotinylation of Sec Body Proteins

**Figure 1:**
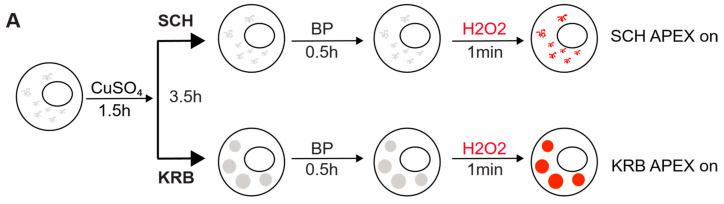

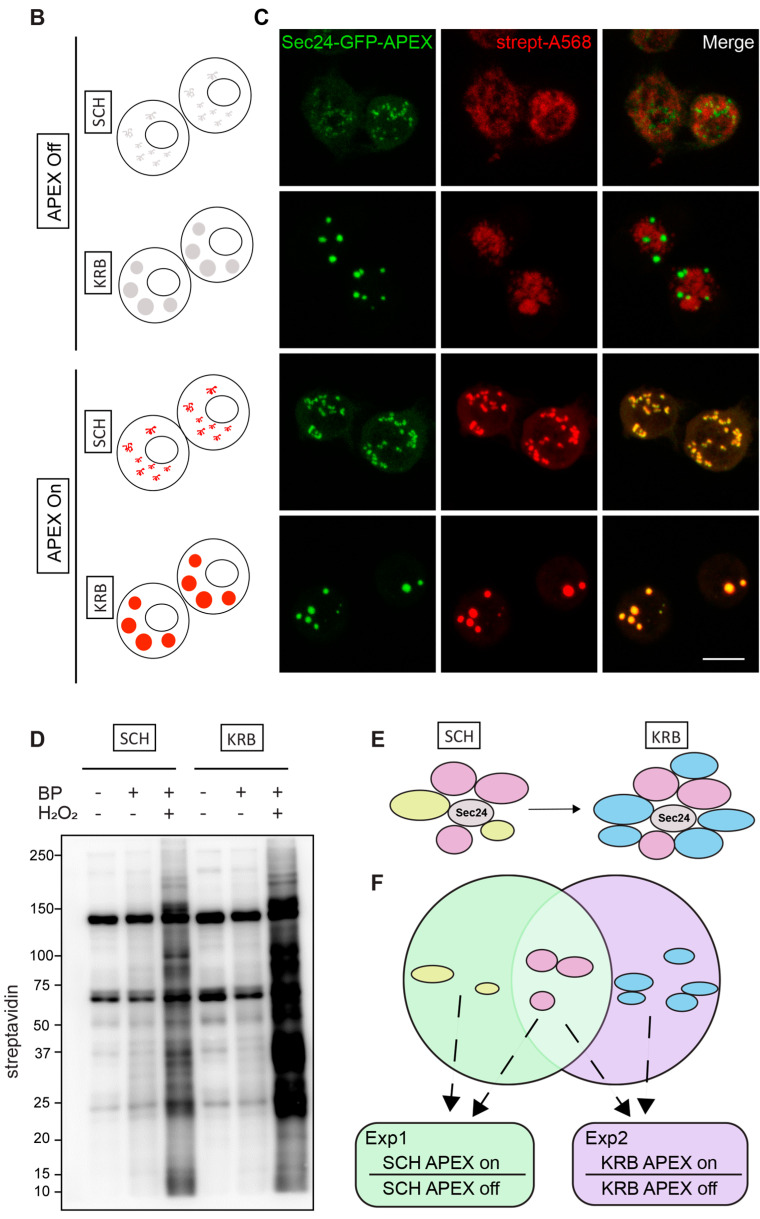
Modeling the Sec24 interactome during growing and starvation conditions. (**A**) Schematic workflow of APEX proximity labeling. For each condition, cells were treated for a total of 4 h after a 1.5 h induction with CuSO_4_. Biotin-phenol (BP) was added in the last 0.5 h, and H_2_O_2_ in the last 1 min for pulsed biotin labeling within the vicinity of Sec24. The biotinylated Sec14AB interactome is marked in red. (**B**) Expected outcome of APEX labeling and Sec body formation. In the absence of APEX triggered by H_2_O_2_, Sec bodies can form upon amino acid starvation buffer KRB incubation, but proteins surrounding Sec24 will not be biotinylated. In the presence of APEX triggered by H_2_O_2_, soluble Sec24 interactors will be labeled by biotin in SCH media, and proteins surrounding Sec24 in starvation-induced Sec bodies will be labeled in KRB media. (**C**) Immunofluorescence image of streptavidin staining in Sec24-APEX2 cells. Complete co-localization of Sec24-GFP-APEX2 and biotin (strept-A568) in Sec bodies in KRB media confirms the specificity of local biotinylation in the Sec24-APEX2 model system. Scale bar: 10 µm. (**D**) Streptavidin-HRP Western blot analysis of induced protein biotinylation in lysates from Sec24-GFP-APEX2 cells. Strong biotinylation signals were detected in the +BP+H_2_O_2_ conditions, where full APEX activation is expected. (**E**) Experimental design to distinguish Sec24 interactors in growing condition and Sec body components during starvation. Soluble Sec24 interactors are annotated in yellow (Exp 1); Sec body proteins during starvation are annotated in blue (Exp 2). (**F**) Universal interactors that may exist in both conditions are annotated in pink (overlap).

To investigate the protein species packaged in Sec bodies, we performed proximity labeling using an inducible ascorbate peroxidase (APEX2) system in the S2 cell line (Figure 1A). To spatially localize the APEX activity to Sec bodies, we fused GFP-APEX2 C-terminally to Sec24, which is a well-characterized Sec body protein [12]. Such a construct allows the fusion protein expression to be monitored by GFP fluorescence, proximity biotin ligation to be assessed by streptavidin-A568 staining, and spatial co-localization of Sec24 and biotinylated Sec body proteins to be verified by an overlay of fluorescence signals in confocal microscopy images. In our model system, not only is the expression of Sec24-GFP-APEX2 inducible, but the APEX activity can be further controlled by the addition or removal of biotin-phenol (BP) and H_2_O_2_, which are critical cofactors essential for biotinylation to take place. The +BP-H_2_O_2_ (APEX off) condition serves as the most stringent control to eliminate background biotinylation events independent of APEX2 (Figure 1B). In the absence of the APEX reaction triggered by H_2_O_2_, Sec bodies can form upon KRB incubation, but proteins surrounding Sec24 will not be biotinylated. In the presence of APEX triggered by H_2_O_2_, soluble Sec24 interactors will be labeled by biotin in SCH media, whereas proteins surrounding Sec24 in starvation-induced Sec bodies will be labeled in KRB media, a condition that readily induces Sec body formation [13].

As shown in Figure 1C, in APEX off condition, the streptavidin staining is diffuse in the cytoplasm. In SCH APEX, the streptavidin staining colocalizes with Sec24 at ER exit sites, consistent with the expected localization of Sec24 in growing conditions [16]. In KRB APEX on condition, Sec24 co-localizes perfectly with local biotinylation in Sec bodies induced by KRB. Biochemically, proximally biotinylated proteins can be prominently retrieved by streptavidin pulldown (Figure 1D), against the background of endogenously biotinylated proteins, such as acetyl-coA carboxylases. With two independent experiments, each activating Sec24-APEX2 under the growing (SCH) or stress (KRB) conditions, we aim to elucidate the protein composition of Sec bodies in a hypothesis-free and unbiased manner (Figure 1E,F) by mass spectrometry.

### 3.2. The Sec24 Spatial Interactome

Since Sec24 is robustly localized to Sec bodies during amino acid starvation in KRB, the interactome proximal to Sec24 under KRB conditions approximates the sub-cellular Sec body proteome, although a physical isolation of such a membrane-less organelle is not yet possible. By virtue of biotin ligation on other proximal proteins in the vicinity of Sec24, it is possible to compare the spatial interactome of Sec24 during growing and starvation conditions, respectively, using our Sec24-GFP-APEX2 inducible system, to identify *bona fide* Sec body proteins. The premise of the investigation is that cytosolic Sec24 will localize at Sec bodies upon starvation in KRB, and hence be surrounded by different proteins that together make up the Sec body (Figure 1E). Sec24-interacting proteins can be (1) specific to growing conditions (Figure 1E, yellow), (2) specific to KRB stress (Figure 1E, blue) or (3) shared in both conditions (Figure 1E, pink). To distinguish between these different types of Sec24 interactors, we employed two experimental schemes, each compared internally to the APEX off condition, to rule out APEX-independent biotinylation (Figure 1F).

**Figure 2:**
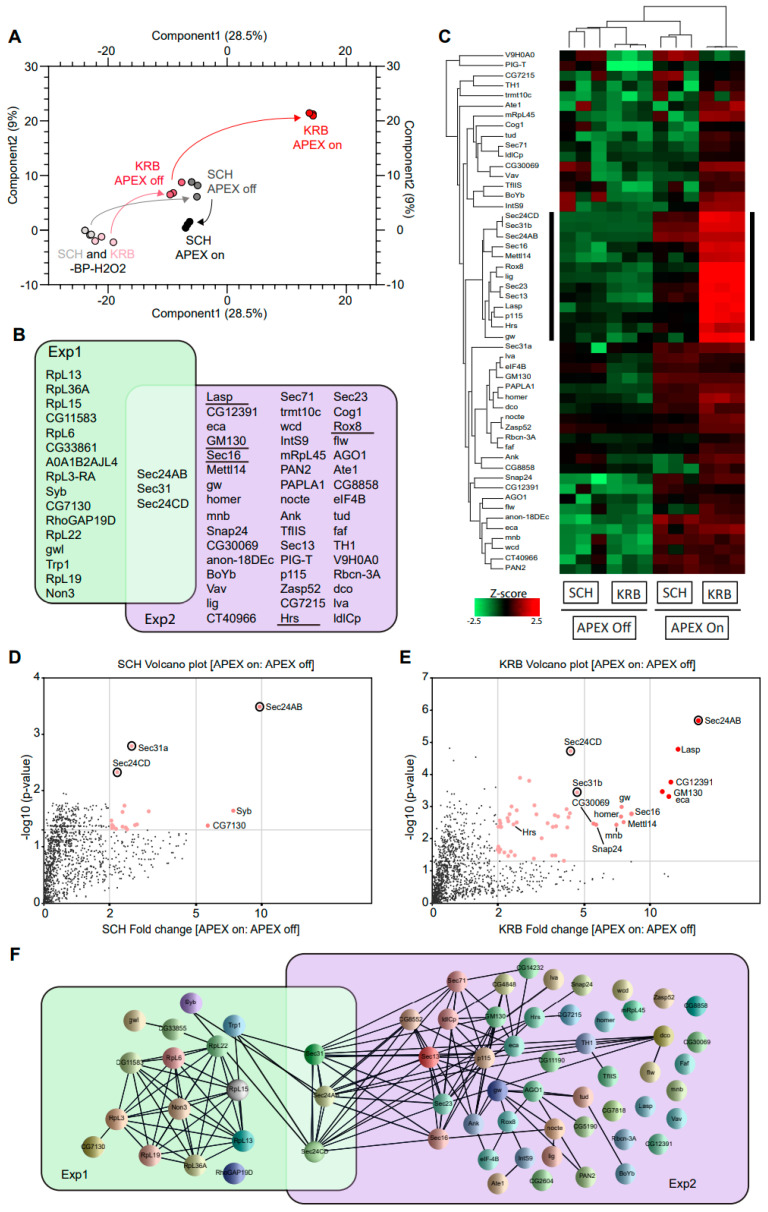
Sec24-APEX2 mediates the biotinylation of Sec body proteins. (**A**) Principal component analysis (PCA) depicting the impact of BP addition and H_2_O_2_ APEX activation. In -BP-H_2_O_2_ conditions, APEX labeling in SCH and KRB have a similarly low background (light grey and light pink, respectively). In +BP-H_2_O_2_ conditions, APEX labeling in SCH and KRB remain similar (dark grey and dark pink, respectively). Differential proximity biotinylation in Sec bodies (red) depends largely on H_2_O_2_. (**B**) Overlap of proteins biotinylated in the vicinity of Sec24. Sec24 has a largely distinct protein interactome during growing (green) and starvation (purple) conditions. Underlined proteins have been experimentally verified to co-localize with Sec bodies in this manuscript. (**C**) Heatmap of differential protein biotinylation in SCH and KRB incubation. Biotinylation is enriched only in APEX on conditions, and preferentially during KRB incubation, where more proteins in Sec bodies are within close proximity to Sec24. Black side bar indicates the most biotinylated 13 proteins in KRB APEX on. (**D**,**E**) Analyses of fold change and significance. Volcano plots feature differentially biotinylated proteins in SCH (**D**) and KRB (**E**) conditions, respectively. (**E**) Box scatter plot showing intrinsically disordered region (IDR) ratio of Sec bodies proteins, corresponding to the 13 core biotinylated proteins indicated by black sidebar in (**C**) and seven of these proteins have a very high IDR (indicated in red). (**F**) Interaction network analysis of biotinylated proteins. Each line represents a documented interaction between biotinylated proteins, extracted from the STRING functional protein association network database.

After cell lysis and streptavidin pulldown, biotinylated proteins were analyzed by quantitative mass spectrometry in triplicates. In Figure 2A, data points of the same color indicate the close technical reproducibility of mass spectrometry measurements, for samples of the same condition. The overall change in biotinylation from -BP-H_2_O_2_ to +BP-H_2_O_2_ (APEX off) to +BP+H_2_O_2_ (APEX on) conditions can be followed in the PCA plot by black arrows (for growing condition) and red arrows (for KRB condition), respectively. The biggest deviation in the path between APEX on and APEX off conditions was observed with KRB, suggesting that the Sec24 interactome shows the biggest change upon KRB stress, and that our experimental setup was highly selective and well-capable of identifying *bona fide* proteins targeted to Sec bodies. An assessment of 2679 proteins identified in total across all conditions, only 19 and 52 proteins (Figure 2B) were specifically enriched in SCH (Exp 1) and KRB (Exp 2) conditions, respectively, using a threshold of at least two-fold quantitative enrichment in APEX on: APEX off (*p* < 0.05). These constitute Sec24 interactors and putative Sec body components, respectively.

The focused heatmap visualization of only putative Sec body proteins (Figure 2C) clearly shows that these 52 proteins are highly APEX-dependent. In addition, a small sub-cluster (annotated with black sidebar) seems to be significantly more enriched in the KRB APEX on condition. Specific Sec24 interactors are quantitatively visualized in Figure 2D,E. Interestingly, the overlap between Exp 1 and Exp 2 is small and consists only of Sec31 and Sec24CD (Figure 2B; circle in Figure 2D,E) that were biotinylated in both growing and KRB conditions. This suggests that these proteins are common interactors in both conditions, regardless of whether Sec24AB is at ERES or in Sec bodies. This finding is consistent with the function of these proteins as COPII subunits in COPII coat formation and their known recruitment to KRB-induced Sec bodies [12].

To further understand the relationship between the other proteins spatially associated with Sec24, we also retrieved all known protein–protein interactions documented amongst Sec24 interactors in growing and KRB conditions, respectively. Extensive reported interactions provide evidence that formation of the Sec body may in part rely on the coalescence of putative complexes forming around COPII subunits (Figure 2F). Interestingly, while we expect COP II components to be extensively biotinylated in SCH, this was not experimentally observed except for Sec31 and Sec24CD. This may be due to the transient nature of ERES complexes not being kinetically favorable for APEX labeling. Empirical detection of several nuclear proteins may also be rationalized with prior reports of such mechanisms of retrograde transport [42,43]. Collectively, these data systematically reveal the protein components in Sec bodies for the first time.

### 3.3. Validation of Sec Body Components Identified by APEX

**Figure 3:**
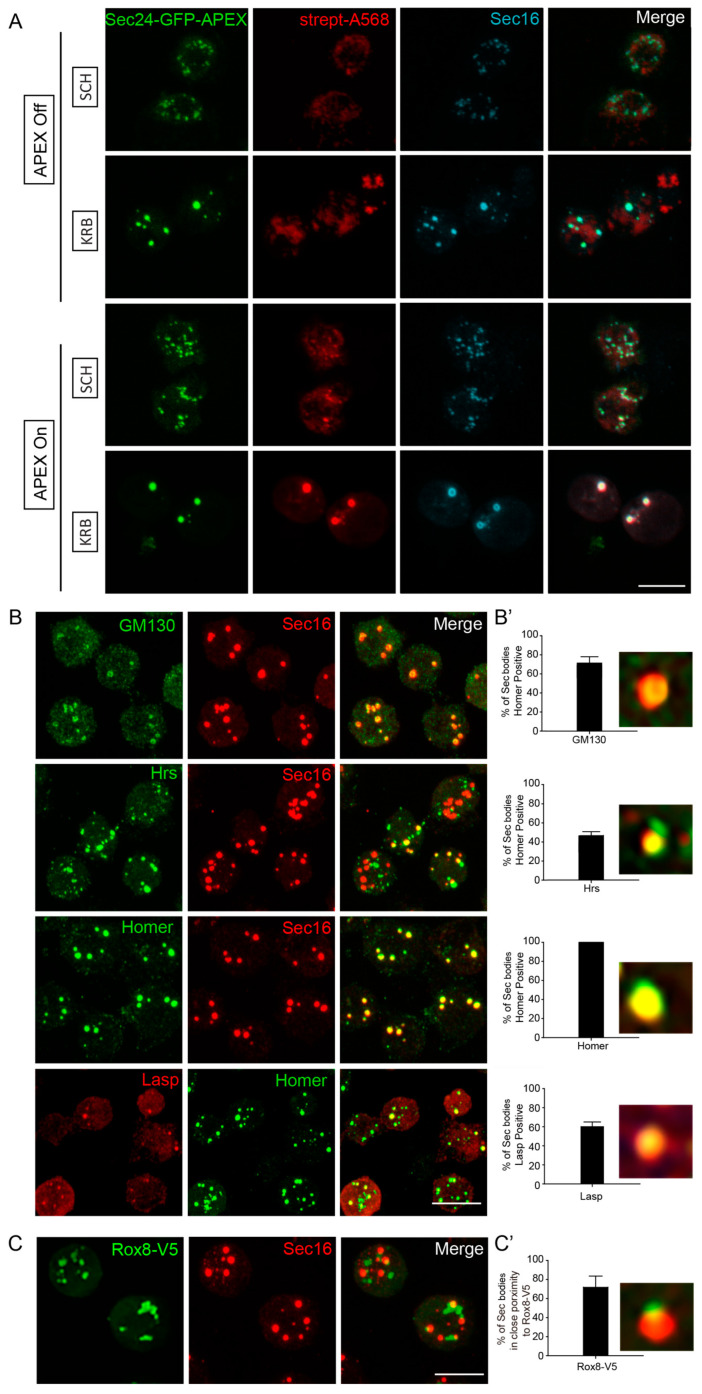
Validation of Sec body proteins by fluorescence confocal microscopy. (**A**) Immunofluorescence visualization of Sec24AB-GFP-APEX2 (green), Sec16 (cyan) and biotin (Strept-A568, red). Sec16 completely co-localizes with Sec24-GFP-APEX2 and biotin. (**B**,**B’**) Immunofluorescence visualization of endogenous GM130, Hrs, Homer (green) in Sec bodies (marked by Sec16, red), as well as Lasp (red) and Homer (green). (**B’**) Percentage of Sec bodies positive for these candidates. Error bars in denote standard deviation in the number of Sec bodies positive for overlap with each target, across 50 image fields. (**C**,**C’**) Immunofluorescence visualization of overexpressed Rox8-V5 (anti V5, green) in close proximity of Sec bodies (marked by Sec16). Percentage of Sec bodies positive for Rox8-V5 (**C’**). Scale bar: 10 µm. Immunofluorescence visualization of putative Sec body proteins and Rox8 in SCH media is shown in Supplementary Figure S1.

To validate that the predicted Sec body components are true components, we used immunofluorescence to assess their co-localization with Sec16, a known Sec body marker [12,13]. Importantly, Sec16 was also on our APEX hit list, and clearly co-localized with Sec24 and biotin (Figure 3A). Co-staining of putative Sec body proteins together with Sec16, instead of Sec24, should further increase confidence, in a manner analogous to reverse IP with a different antibody. Using this strategy, we verified the presence of newly identified components of KRB-induced Sec bodies (Figure 3B). Endogenous GM130 (a Golgi peripheral protein) [44], Hrs (an endosome-associated protein) [45], Homer (a scaffold protein at postsynaptic density) [46] and Lasp (an actin-associated protein) [47] significantly localized to KRB-induced Sec bodies marked with Sec16 (Figure 3B,B’). Interestingly, not all the validated Sec proteins were present in every observed Sec body, as shown in Figure 3B’. This reveals that the packaging of proteins into Sec bodies may be somewhat heterogeneous and not always coordinated in component stoichiometry. This is also a key characteristic of assemblies formed by phase separation.

In addition, V5-tagged Rox8, the Drosophila homologue of TIA1, an established stress granule marker in mammalian cells [48] was found in close proximity to Sec bodies (Figure 3C,C’). This is in agreement with the earlier observation that stress granules form close to Sec bodies [12] and share components [11]. The validations of these five candidates further strengthen the reliability of our mass spectrometry approach, and for the first time, we can systematically identify the protein components of KRB-induced Sec bodies.

### 3.4. Sec Bodies Form from the Re-Localization of Existing Intracellular Proteins

**Figure 4:**
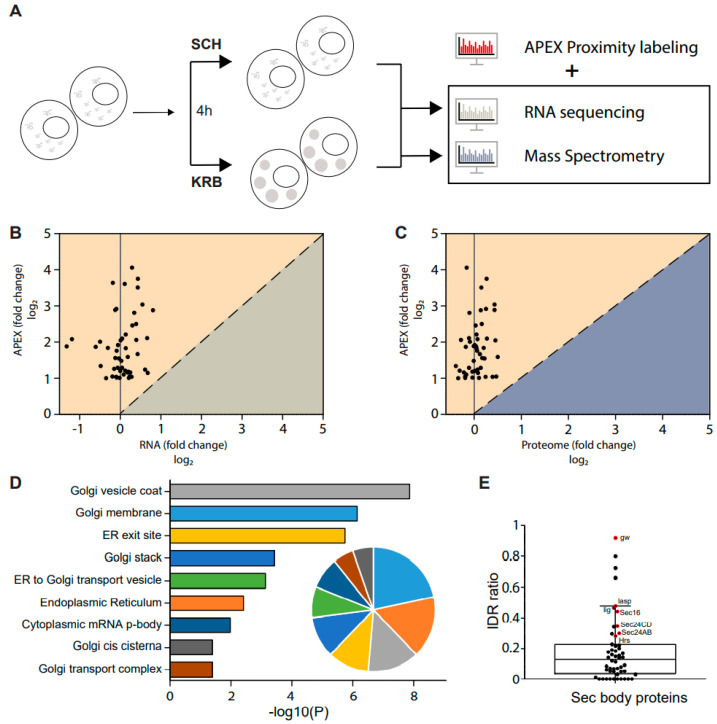
Sec body formation depends on the re-localization of existing cytosolic proteins. (**A**) Schematic of combined RNA, protein and APEX analyses of KRB-treated cells. Differential changes at 4 h starvation, if any, were analyzed for correlative trends. (**B**,**C**) Correlation between significantly enriched Sec body proteins and the respective transcript levels (**B**), or total proteome levels (**C**). Almost all proteins that accumulate in Sec bodies were not differential at the RNA or total proteome level. (**D**) Bar graph depicting significantly enriched gene ontology cellular compartment (GO/CC) terms for proteins in Sec bodies, as calculated by DAVID. The pie graph inset shows the relative proportion of Sec body proteins annotated to these cellular compartments. A large majority of Sec body proteins are of ER and Golgi origin. (**E**) Box scatter plot showing 52 Sec body proteins ranked by the proportion of intrinsically disordered region (IDR). Proteins annotated in red were significantly enriched in Sec bodies, as indicated by black side bar in the heatmap in Figure 2C.

Since the observation of Sec bodies, the mechanism surrounding the formation of their stress-induced assemblies has remained largely speculative. Of note, specific questions regarding the origin of proteins packaged in these compartments remain unanswered. To investigate this, we supplemented the APEX data with both RNA sequencing (GSE143810 [13] and total proteome data acquired here, under the same conditions (Figure 4A). Since the total cellular proteome considers all proteins, inclusive of proteins packaged into Sec bodies, it is possible to determine if the total quantity of each putative Sec body protein inside the cell has increased during the KRB stress. As shown in Figure 4B,C, none of the putative Sec body proteins were up-regulated at the RNA or proteome level. This provides strong evidence that proteins packaged in Sec bodies are neither newly transcribed nor newly translated. Instead, Sec bodies are likely to be re-organized from existing intracellular protein pools.

Detailed gene ontology cellular component analysis on the putative Sec body proteins identified in our dataset further revealed that these proteins are largely localized to the ER and Golgi under normal growth conditions (Figure 4D), thereby providing further support that Sec bodies may form from the re-organization of peripheral proteins functioning at the early secretory pathway.

While other types of stress-induced assemblies, such as stress granules, have been studied more extensively and also by a similar APEX approach we have used here [34], the same gene ontology cellular compartment analysis of stress granule components revealed drastically different sub-cellular enrichments, for instance, predominantly in the nuclear and cytoplasmic compartments, and were not membrane-associated (Supplementary Table S1). Furthermore, stress granules reportedly contain hundreds of different proteins from diverse subcellular compartments, whereas Sec bodies are composed of relatively fewer proteins that are mostly of ER and Golgi origin. These differences critically distinguish their formation.

Sec bodies have the properties of phase-separated liquid droplets [12] similar to stress granules. The components of phase-separated liquid droplets have been reported to often contain intrinsically disordered regions (IDRs) [49] that may facilitate droplet formation. Many IDRs contain low-complexity domains (LCDs), which are regions with low amino acid diversity [50,51]. We have previously established that Sec24AB/CD and Sec16 display a high LCD content [12]. Uniprot sequence annotation of the 52 putative Sec body proteins identified in this current dataset revealed a wide range of IDR ratios, as defined by the fraction of the total sequence length predicted to be intrinsically disordered. It was intriguing to find that the cluster of proteins most enriched in Sec bodies (Figure 2C, black side bars) are also amongst the proteins with the highest IDR ratio in our dataset (Figure 4E, data points in red). In addition, 42 out of the 52 putative Sec body proteins contain IDRs (Figure 4E). This further implies that Sec bodies may form from the coalescence of ERES, ER and Golgi components as driven by the lattice arrangement between intrinsically disordered regions of driver proteins, such as Sec16 and Sec24AB at ERES. These can, in turn, nucleate the other proteins that function normally in the early secretory pathway.

### 3.5. Concluding Remarks

Collectively, these observations critically suggest that Sec bodies are distinct from other stress granules in both the triggers for formation, as well as in composition. Regarding the latter, KRB stress imposes, in part, an amino acid starvation condition that limits raw materials for protein synthesis. We speculate that this renders the secretory pathway temporarily irrelevant, as membrane traffic through the ER and predominantly the Golgi apparatus is temporarily halted. In the face of disuse, ER and Golgi protein machinery seem to be placed into storage and re-organized into Sec bodies via phase separation through the initial nucleation of proteins with intrinsically disordered regions. This would preserve the ER and Golgi machinery while awaiting the return to favorable conditions for protein biosynthesis. The reversible phase separation of ER and Golgi proteins that we identify here may, in turn, allow for the coordinated regulation of the entire secretory pathway.

## Data Availability

The data presented in this study are available within the article. For any further request contact the corresponding author.

## Supplementary Materials

The following supporting information can be downloaded at: https://www.mdpi.com/article/10.3390/cells12071055/s1, Table S1: Gene ontology cellular compartment analysis of stress granule components, as calculated by DAVID; Figure S1. (A) Immunofluorescence of GM130, Hrs, Homer (green) in Sec bodies (marked by Sec 16, red), as well as Homer (green) and Lasp (red), in SCH media under growing conditions. (B) Immunofluorescence of V5-tagged Rox8 in SCH media.
