# Supplementary material for: Novel Components of the Stress Assembly Sec Body Identified by Proximity Labeling
Source: Cells. 2023 Mar 30;12(7):1055. doi: 10.3390/cells12071055 (PMC10093351; doi:10.3390/cells12071055)
Supplement: Supplementary file 1 [file cells-12-01055-s001.zip › cells-2211308-supplementary.pdf]

Supplementary Figure S1

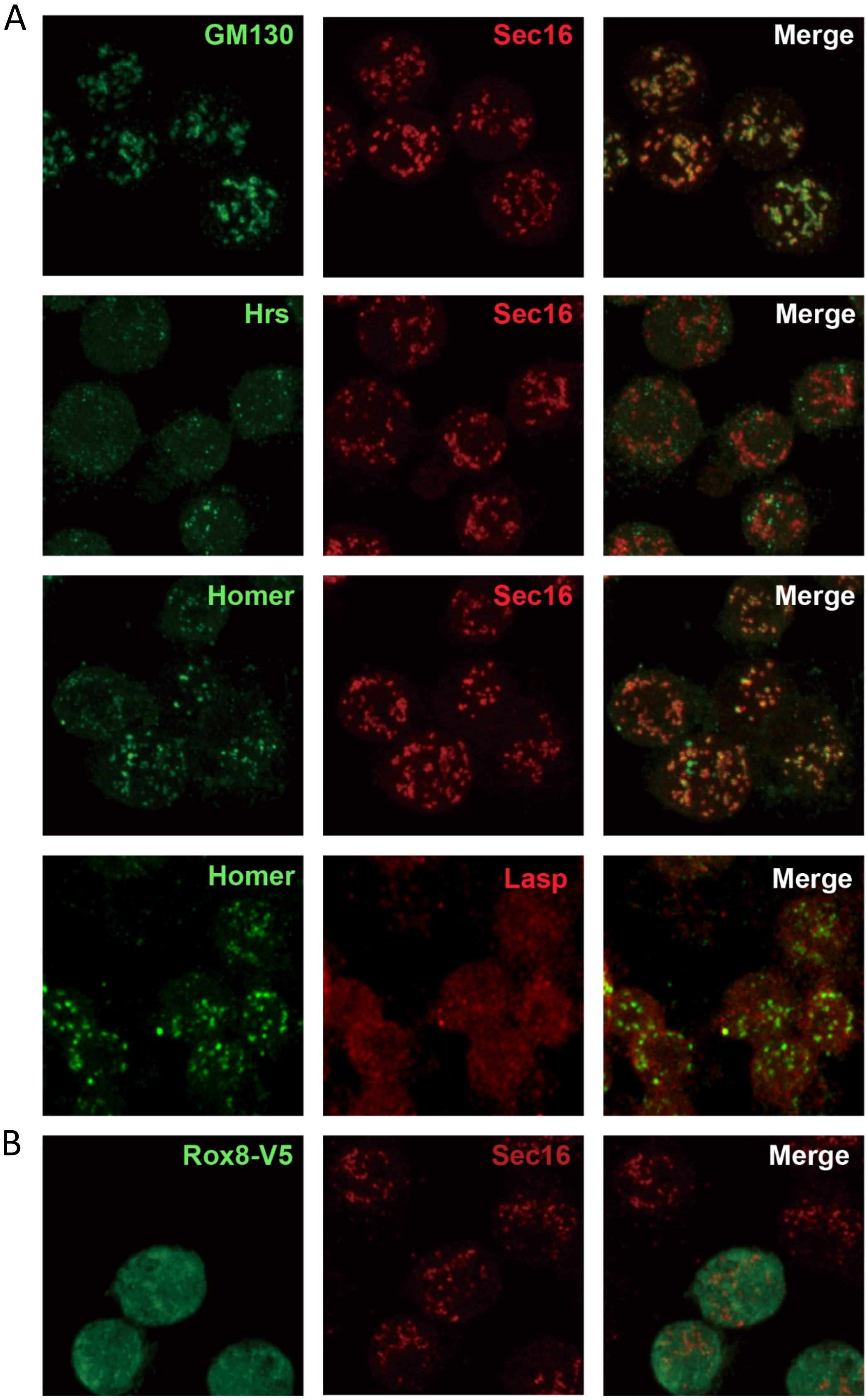

Supplementary Figure S1. (A) Immunofluorescence of GM130, Hrs, Homer (green) in Sec bodies (marked by Sec 16, red), as well as Homer (green) and Lasp (red), in SCH media under growing conditions. (B) Immunofluorescence of V5-tagged Rox8 in SCH media.

Supplementary Table S1

Gene Ontology: Cellular Components for Stress Granules												
**Category**	**Term**	**Protein Name**	**%**	**PValue**	**Genes**	**List Total**	**Pop Hits**	**Pop Total**	**Fold Enrichment**	**Bonferroni**	**Benjamini**	**FDR**
GOTERM_CC_DIRECT	GO:0030529	intracellular ribonucleoprotein complex	15.4839	8.96E-24	Q53F48, B7Z645, F8W646, A0A024R7T3, A0A087WTT1, F8W0G4, H7C1J8, H3BR27, B1ANR0, Q59ES8, E5RIZ6, A0A087X1I2, A0A087WU03, Q5TBP9, B4DY08, Q5HYY5, B4DMD1, A0A087WUI2, K7EQ75, Q53FG3, A4FVC0, E9PQK4. A0A087WXI3. C9J5V9	154	136	18224	20.88311688	1.96E-21	1.96E-21	1.84E-21
GOTERM_CC_DIRECT	GO:0010494	cytoplasmic stress granule	9.67742	1.51E-20	Q8IV96, I3L3A7, D6W5Y5, A0A087WTT1, A0A087WX93, C9JE24, B1ANR0, E5RIZ6, A1L3A7, Q5T1Z4, A0A0G2JQ95, C9JTN7, B4DLA0, C9J5V9, A0A0C4DGL7	154	36	18224	49.30735931	3.30E-18	1.65E-18	1.55E-18
GOTERM_CC_DIRECT	GO:0005634	nucleus	64.5161	2.29E-19	E9PIW1, Q53F48, H3BPE7, A0A024R1N2, Q5SY38, A1L3A7, J3KSR8, B4E0E6, E9PJ07, B4DY08, J3KS93, D6R9T8, A0A075B6F9, E9PNQ0, H3BNJ0, A0A087WXM7, Q05DU1, Q5JR91, A0PJM7, F8VZR4, I3L382, K7EKV2, A0A087WU03, E2QRN4, Q9H5V0, H0YHL2, H0YH33, S4R446, A0A024R7T3, I3L3A7, A2RUM0, X5C404, A8K503, F8W0G4, A0A087WUI2, B4DLA0, A4FVC0, Q8N4U2, B1ALY4, A0A087WTY4, H7C1J8, K7EKX4, A0A087X1I2, H7BZN3, F5GXJ8, C9K0E5, E9PQ98, H0YLV8, H7C4Y3, E9PJL7, L0R5B6, E7EQJ0, B7Z645, F8W646, D6W5Y5, L0R4Y0, A0A087WX93, H3BR53, A0A075B7D6, A0A0D9SEI7, D3DWF3, A0A087WXI3, E9PQK4, H7C4Z9, F8WEA1, A8K3N4, W0HJ78, C9JF56, Q5QPM1, H7C4B6, Q53FG3, B4DT31, B0QZE6, X5DP08, A0A087X0R2, C9JMM0, A0A087WTT1, A0A024RAC5, H3BR27, B1ANR0, C9JG86, A0A087WVP1, I3L0P0, F5GYI9, E7EPF2, L8EBD0, F8WD15, J3QLH2, Q5JRC6, Q8IV96, F8W930, D6RCL3, A6NG75, E5RIZ6, B7Z2D6, E9PP32, K7EQ75, B1AMN9, C9J5V9, D6RCD8	154	5415	18224	2.185367726	5.02E-17	1.67E-17	1.57E-17
GOTERM_CC_DIRECT	GO:0005654	nucleoplasm	41.2903	8.50E-15	Q53F48, L0R5B6, H3BPE7, E7EQJ0, B7Z645, F8W646, D6W5Y5, A0A0D9SG84, A0A087WX93, J3KSR8, B4E0E6, B4DY08, I3L218, Q5HYY5, A0A075B7D6, H3BNJ0, D3DWF3, A0A087WXI3, E9PQK4, F8WEA1, A8K3N4, A0A0A0MR72, A0A087WU03, E2QRN4, F8VWK9, Q9H5V0, H0YHL2, Q53FG3, B4DT31, F5H1L3, B0QZE6, A0A024R7T3, A0A087X0R2, A2RUM0, E9PRF1, X5C404, A8K503, F8W0G4, H3BR27, C9JG86, Q59ES8, H0Y8W1, F5GYI9, S4R3G0, C9JTN7, J3QLH2, A0A087WUI2, C9JRJ8, A4FVC0, Q5JRC6, D6RCL3, A0A087WTY4, H7C1J8, K7EKX4, A0A087X1I2, H7BZN3, C9K0E5, F5GXJ8, E9PQ98, B4DMD1, K7EQ75, H0YLV8, E9PJL7, C9J5V9	154	2784	18224	2.720406031	1.87E-12	4.65E-13	4.35E-13
GOTERM_CC_DIRECT	GO:0071013	catalytic step 2 spliceosome	9.03226	7.76E-13	E7EQJ0, B7Z645, F8W646, A0A024R7T3, A0A087WTT1, H7C1J8, H3BR27, Q59ES8, Q5QPM1, F5GYI9, J3KSR8, B4DY08, B4DMD1, A0A087WUI2	154	92	18224	18.00790514	1.70E-10	3.40E-11	3.18E-11
GOTERM_CC_DIRECT	GO:0019013	viral nucleocapsid	6.45161	9.85E-13	Q53F48, B7Z645, A0A024R7T3, F8W646, B4DY08, B4DMD1, H7C1J8, Q59ES8, A0A087X1I2, A0A087WU03	154	28	18224	42.26345083	2.16E-10	3.60E-11	3.37E-11
GOTERM_CC_DIRECT	GO:0005737	cytoplasm	54.8387	8.12E-12	E9PIW1, E7EQJ0, F8W646, A0A0S2Z415, Q5SY38, D6W5Y5, L0R4Y0, A0A087WX93, C9JE24, A1L3A7, J3KSR8, A0A0G2JQ95, B4E0E6, Q8IWJ7, J3KS93, C9K0B6, A0A075B7D6, F8VXB3, A0A075B6F9, E9PNQ0, A0A0D9SEI7, D3DWF3, A0A087WXM7, A0A087WXI3, E9PQK4, Q05DU1, F8WEA1, C9JLV6, A0A0D9SF19, K7EKV2, Q5VU79, A0A087WU03, F8VWK9, Q9H5V0, H0YE19, H0YHL2, H7C4B6, Q53FG3, C9JY20, A0A024R3V0, H0YH33, B7ZM29, B0QZE6, A0A024R7T3, X5DP08, I3L3A7, A2RUM0, X5C404, A0A087WTT1, A8K503, F8W0G4, B1ANR0, H0Y8W1, L8EBD0, F8WD15, C9JTN7, A0A087WUI2, B4DLA0, C9JRJ8, A4FVC0, S4R373, F8W930, Q8IV96, D6RCL3, A0A087WTY4, H7C1J8, A6NJA2, E9PLT0, E5RIZ6, L8EA03, H7BZN3, C9J9B2, B7Z2D6, C9K0E5, F5GXJ8, E9PQ98, K7EQ75, A0A087WVH0, H0YLV8, B1AMN9, H7C4Y3, A6NEP8, E9PJL7, A0A0C4DGL7, C9J5V9	154	5222	18224	1.926216258	1.78E-09	2.54E-10	2.38E-10
GOTERM_CC_DIRECT	GO:0005730	nucleolus	17.4194	1.16E-08	L0R5B6, A2RUM0, E5RFK1, D6W5Y5, A0A024RAC5, Q59ES8, S4R3G0, B4E0E6, A0A075B7D6, J3QLH2, D3DWF3, Q5JRC6, A0A087WXI3, Q05DU1, Q8IV96, D6RCL3, K7EKX4, K7EKV2, F5GXJ8, Q9H5V0, B4DMD1, K7EQ75, H0YLV8, H0YHL2. Q53FG3. C9JY20. A0A024R3V0	154	857	18224	3.72825774	2.53E-06	3.16E-07	2.96E-07
GOTERM_CC_DIRECT	GO:0016020	membrane	27.0968	5.18E-07	A0A075BTL2, E7EQJ0, B7Z645, F8W646, A0A024R7T3, X5DP08, Q5BKY2, A0A0S2Z415, E5RFK1, A0A087WTT1, A0A024RAC5, L0R4Y0, F8W0G4, H3BR27, Q59ES8, A0A087WVP1, I3L0P0, A1L3A7, L8EBD0, Q8IWJ7, B4DY08, J3KS93, A0A087WUI2, A4FVC0, A0A087WXI3, E9PQK4, Q05DU1, Q8IV96, Q5JR91, H7C5N8, K7EKX4, C9K0E5, H3BSQ0, E9PP32, K7EQ75, F8VNV1, Q53FG3, C9JY20, H7C4Y3, A6NEP8, A0A0C4DGL7, H0Y8H5	154	2200	18224	2.259173554	1.14E-04	1.26E-05	1.18E-05
GOTERM_CC_DIRECT	GO:0005681	spliceosomal complex	5.16129	1.42E-05	Q53F48, E2QRN4, F8W646, B4DY08, B4DMD1, A0A087WUI2, Q59ES8, A0A087WU03	154	94	18224	10.07129041	0.00311273	3.12E-04	2.92E-04
GOTERM_CC_DIRECT	GO:0005844	polysome	3.87097	1.57E-05	H3BPE7, F8VX11, L0R4Y0, A4FVC0, C9JY20, A0A087WXI3	154	38	18224	18.68489405	0.00342809	3.12E-04	2.92E-04
GOTERM_CC_DIRECT	GO:0000932	cytoplasmic mRNA processing body	4.51613	4.95E-05	A0A0G2JQ95, Q5T1Z4, H3BSQ0, Q8IV96, I3L218, C9JRJ8, A4FVC0	154	78	18224	10.62004662	0.01079206	9.04E-04	8.46E-04
GOTERM_CC_DIRECT	GO:0005829	cytosol	30.9677	1.13E-04	E9PIW1, A0A075BTL2, X5DP08, Q5BKY2, A0A0S2Z415, A0A087WTT1, A0A024RAC5, E9PRF1, A8K503, F8W0G4, H0YED1, I3L0P0, H3BMT9, Q8IWJ7, F8WD15, B4DY08, J3KS93, I3L218, A0A075B6F9, C9JRJ8, D3DWF3, H3BNJ0, A4FVC0, A0A087WXM7, F8W930, Q8IV96, C9JLV6, Q5JR91, A0A087WTY4, F8VX11, A6NG75, C9JF56, A0A0D9SF19, E5RIZ6, K7EKV2, B7Z2D6, F8VWK9, C9K0E5, H3BSQ0, Q5T1Z4, E9PQ98, E9PP32, Q5TFK1, H7C4B6, A6NEP8, H0YH33, E9PJL7, H0Y8H5	154	3315	18224	1.713486513	0.02445933	0.00179988	0.00168481
GOTERM_CC_DIRECT	GO:0000775	chromosome, centromeric region	3.87097	1.15E-04	Q5JR91, E9PP32, A0A087WTY4, C9JMM0, A0A087WXI3, I3L0P0	154	57	18224	12.45659604	0.02488484	0.00179988	0.00168481
GOTERM_CC_DIRECT	GO:0005694	chromosome	4.51613	2.58E-04	B4E0E6, B0QZE6, A0A087X0R2, J3QLH2, A0A087WXI3, C9JG86, H0Y8W1	154	105	18224	7.889177489	0.05490673	0.00376429	0.00352365
GOTERM_CC_DIRECT	GO:0070937	CRD-mediated mRNA stability complex	1.93548	0.00103	B7Z645, E9PLT0, C9J5V9	154	6	18224	59.16883117	0.20158035	0.01406283	0.01316384
GOTERM_CC_DIRECT	GO:0005852	eukaryotic translation initiation factor 3 complex	1.93548	0.00877	Q5BKY2, B4DLA0, Q5TFK1	154	17	18224	20.88311688	0.85464927	0.11294932	0.10572882
GOTERM_CC_DIRECT	GO:0005697	telomerase holoenzyme complex	1.93548	0.01324	B4DY08, Q05DU1, K7EKV2	154	21	18224	16.90538033	0.94606767	0.16114691	0.15084528
GOTERM_CC_DIRECT	GO:0035145	exon-exon junction complex	1.93548	0.01449	B1AMN9, K7EKV2, J3KSR8	154	22	18224	16.13695396	0.95909564	0.16701597	0.15633915
GOTERM_CC_DIRECT	GO:0043231	intracellular membrane-bounded organelle	7.09677	0.01949	L8EBD0, A0A0G2JQ95, E2QRN4, Q8IV96, F8VWK9, B4E0E6, B0QZE6, I3L218, X5C404, H0Y8H5, C9J5V9	154	558	18224	2.332821301	0.98655828	0.21336346	0.19972378
GOTERM_CC_DIRECT	GO:0000776	kinetochore	2.58065	0.03076	Q5JR91, A0A087WTY4, D3DWF3, I3L0P0	154	81	18224	5.843835177	0.9989332	0.31578123	0.2955943
GOTERM_CC_DIRECT	GO:0000942	condensed nuclear chromosome outer kinetochore	1.29032	0.03316	D3DWF3, I3L0P0	154	4	18224	59.16883117	0.99938021	0.31578123	0.2955943
GOTERM_CC_DIRECT	GO:0031262	Ndc80 complex	1.29032	0.03316	E9PP32, I3L0P0	154	4	18224	59.16883117	0.99938021	0.31578123	0.2955943
GOTERM_CC_DIRECT	GO:0048471	perinuclear region of cytoplasm	7.09677	0.03683	F5H1L3, S4R446, H3BPE7, X5DP08, A0A087WTY4, C9JY20, C9JE24, A0A087WXI3. A0A087WVP1. A6NEP8. H0Y8H5	154	621	18224	2.09615827	0.99973001	0.32296659	0.30232033
GOTERM_CC_DIRECT	GO:0000777	condensed chromosome kinetochore	2.58065	0.03687	Q5JR91, E9PP32, Q5JRC6, I3L0P0	154	87	18224	5.440812062	0.9997326	0.32296659	0.30232033
GOTERM_CC_DIRECT	GO:0000785	chromatin	2.58065	0.03903	A0A087WTY4, C9JMM0, E9PNQ0, D3DWF3	154	89	18224	5.318546622	0.99983657	0.32876197	0.30774522
GOTERM_CC_DIRECT	GO:0016363	nuclear matrix	2.58065	0.04831	E2QRN4, A0A087WTY4, H0YLV8, Q59ES8	154	97	18224	4.879903602	0.99998048	0.37255864	0.34874211
GOTERM_CC_DIRECT	GO:0035068	micro-ribonucleoprotein complex	1.29032	0.04933	I3L218, A4FVC0	154	6	18224	39.44588745	0.99998458	0.37255864	0.34874211
GOTERM_CC_DIRECT	GO:0071204	histone pre-mRNA 3'end processing complex	1.29032	0.04933	B7Z645, C9J5V9	154	6	18224	39.44588745	0.99998458	0.37255864	0.34874211
GOTERM_CC_DIRECT	GO:0000781	chromosome, telomeric region	1.93548	0.057	H0YLV8, K7EKV2, H7BZN3	154	46	18224	7.717673631	0.99999738	0.39227153	0.36719481
GOTERM_CC_DIRECT	GO:0042788	polysomal ribosome	1.29032	0.05732	A0A087WXI3, A1L3A7	154	7	18224	33.81076067	0.99999757	0.39227153	0.36719481
GOTERM_CC_DIRECT	GO:0071541	eukaryotic translation initiation factor 3 complex, eIF3m	1.29032	0.05732	Q5BKY2, Q5TFK1	154	7	18224	33.81076067	0.99999757	0.39227153	0.36719481
GOTERM_CC_DIRECT	GO:0005813	centrosome	5.16129	0.06819	C9JWZ1, F5GXJ8, B4E0E6, Q8IWJ7, C9JLV6, A0A087WTY4, D3DWF3, A0PJM7	154	426	18224	2.222303518	0.99999981	0.45253753	0.42360819
GOTERM_CC_DIRECT	GO:0016281	eukaryotic translation initiation factor 4F complex	1.29032	0.07309	J3KS93, F8VX11	154	9	18224	26.2972583	0.99999994	0.47076526	0.44067067
GOTERM_CC_DIRECT	GO:0035770	ribonucleoprotein granule	1.29032	0.08087	H7C1J8, C9JY20	154	10	18224	23.66753247	0.99999999	0.49789998	0.46607076
GOTERM_CC_DIRECT	GO:0005819	spindle	2.58065	0.08185	A0A087WTY4, C9JMM0, D3DWF3, H7BZN3	154	121	18224	3.911988838	0.99999999	0.49789998	0.46607076
GOTERM_CC_DIRECT	GO:0016442	RISC complex	1.29032	0.08859	Q8IV96, A4FVC0	154	11	18224	21.51593861	1	0.52437715	0.49085532
GOTERM_CC_DIRECT	GO:0005739	mitochondrion	10.9677	0.09307	Q8IV96, C9JLV6, A0A0S2Z415, A0A0D9SG84, H0YFD6, E5RIZ4, C9K0E5, Q5HYY5, C9K0B6, A0A087WVH0, K7EQ75, A0A0D9SEI7, Q8N4U2, A6NEP8, Q05DU1. E9PJL7. D6RCD8	154	1331	18224	1.511450233	1	0.52696741	0.49328
GOTERM_CC_DIRECT	GO:0030496	midbody	2.58065	0.09477	F5GXJ8, A0A087WTY4, A0A024RAC5, D3DWF3	154	129	18224	3.669384879	1	0.52696741	0.49328
GOTERM_CC_DIRECT	GO:0005845	mRNA cap binding complex	1.29032	0.09625	A4FVC0, A0A087WXI3	154	12	18224	19.72294372	1	0.52696741	0.49328
**Gene Ontology: Cellular Components for Sec Bodies**												
**Category**	**Term**	**Protein Name**	**%**	**PValue**	**Genes**	**List Total**	**Pop Hits**	**Pop Total**	**Fold Enrichment**	**Bonferroni**	**Benjamini**	**FDR**
GOTERM_CC_DIRECT	GO:0017119	Golgi transport complex	4.08	0.04056	FBGN0037998, FBGN0026634	47	9	10026	47.40425532	0.96202902	0.35601587	0.34249628
GOTERM_CC_DIRECT	GO:0000137	Golgi cis cisterna	4.08	0.04056	FBGN0029688, FBGN0034697	47	9	10026	47.40425532	0.96202902	0.35601587	0.34249628
GOTERM_CC_DIRECT	GO:0030134	ER to Golgi transport vesicle	6.12	7.26E-04	FBGN0031990, FBGN0033339, FBGN0262125	47	9	10026	71.10638298	0.05578773	0.01147669	0.01104086
GOTERM_CC_DIRECT	GO:0000932	cytoplasmic mRNA processing body	6.12	0.01052	FBGN0033352, FBGN0051992, FBGN0020279	47	34	10026	18.82227785	0.56647265	0.11877067	0.11426039
GOTERM_CC_DIRECT	GO:0070971	endoplasmic reticulum exit site	8.16	1.79E-06	FBGN0052654, FBGN0262126, FBGN0033339, FBGN0262125	47	6	10026	142.212766	1.41E-04	4.72E-05	4.54E-05
GOTERM_CC_DIRECT	GO:0005795	Golgi stack	8.16	3.71E-04	FBGN0040087, FBGN0034697, FBGN0033339, FBGN0262125	47	31	10026	27.52505148	0.02891574	0.00733415	0.00705564
GOTERM_CC_DIRECT	GO:0030127	COPII vesicle coat	10.20	1.34E-08	FBGN0024509, FBGN0033460, FBGN0262126, FBGN0033339, FBGN0262125	47	7	10026	152.3708207	1.06E-06	1.06E-06	1.02E-06
GOTERM_CC_DIRECT	GO:0005783	endoplasmic reticulum	12.24	0.00388	FBGN0040087, FBGN0030035, FBGN0034697, FBGN0025777, FBGN0033339, FBGN0262125	47	230	10026	5.564847364	0.26420796	0.05103546	0.0490974
GOTERM_CC_DIRECT	GO:0000139	Golgi membrane	16.33	7.14E-07	FBGN0052654, FBGN0024509, FBGN0033460, FBGN0040087, FBGN0262126, FBGN0037998. FBGN0026634. FBGN0033339	47	113	10026	15.10224063	5.64E-05	2.82E-05	2.71E-05
